# Epidermal Growth Factor Downregulates Carbon Anhydrase III (CAIII) in Colon Cancer

**DOI:** 10.3390/cimb46110774

**Published:** 2024-11-14

**Authors:** Derya Okuyan

**Affiliations:** Department of Veterinary Medicine, Susurluk Agriculture and Forestry Vocational School, Bandırma Onyedi Eylül University, Susurluk 10600, Balıkesir, Türkiye; dokuyan@bandirma.edu.tr

**Keywords:** carbonic anhydrase III, CAIII, colon carcinoma, EGF, HT-29, SW480, HUVEC

## Abstract

Colorectal cancer (CRC) is the second leading cause of cancer-related death in the world. Dysregulations in the EGF signaling pathway have been associated with colon cancer. Some members of the carbonic anhydrase family serve as biomarkers in cancer. Carbonic anhydrase III (CAIII), a member of this family, shows different activities than the other members of its family and has been associated with cancer. However, there are no studies on the effective regulation of EGF. In this study, we investigated the EGF-influenced regulation of CAIII in the HT29, SW480, and HUVEC cell lines and showed that CAIII regulation decreased with the effect of EGF. We aimed to investigate the EGF-affected mRNA and protein regulation of the CAIII gene in HT29, SW480, and HUVEC cell lines. For this purpose, we determined time-dependent CAIII mRNA and protein expression by applying EGF to HT29, SW480, and HUVEC cells. Time-dependent EGF-induced mRNA and protein level regulation of the CAIII gene decreased in the HT29, SW480, and HUVEC cell lines. EGF regulates the motility, adhesion, and metastasis of cancer cells. CAIII prevents cells from metastasizing through cell acidification. Therefore, our findings explained why the EGF-effective regulation of CAIII decreased. We suggest that the CAIII gene is promising as a targeted therapy due to the decrease in EGF-effected CAIII gene regulation in colon carcinoma.

## 1. Introduction

Colorectal cancer (CRC) was the second leading cause of cancer-related death worldwide in 2022. Liver metastasis is common in CRC, which has a high mortality rate [1,2,3]. Within three years of diagnosis, almost one-third of patients may experience liver metastases [4,5]. Hematogenous diffusion through portal circulation is the distribution method used [6,7,8,9].

Dysregulation of the EGF receptor (EGFR) signaling pathway is a typical observation in malignancies, especially colorectal cancer (CRC) [10]. The human EGFR (HER)-erbB family of tyrosine kinase receptors (RTKs) comprise the EGFR (ERB-1 or HER-1) as well as three other members, namely HER3 (ErbB3), HER4 (ErbB4), and HER2/C-neu (ErbB2) [11]. As a member of the RTK ErbB family, the glycoprotein EGFR has an external ligand-binding domain in addition to an intracellular tyrosine kinase domain. This ligand binds to the extracellular domain, causing homo- or hetero-dimerization. This process phosphorylates the tyrosine kinase domain and activates the RAS–RAF–MAPK signaling cascade, which in turn promotes the development and spread of tumors [12]. Since EGFR is overexpressed in a variety of malignancies, including colorectal cancer (CRC), it is a great candidate for targeted cancer treatment [13,14]. Furthermore, EGF stimulates the PI3K/AKT/mTOR signaling pathway, which is essential for cell invasion, motility, and survival, upon binding to its receptor, EGFR [12,15]. Furthermore, EGFR mutations are uncommon in CRC [15].

Carbonate dehydrates, or CAs, are common zinc-containing metalloenzymes that reversibly catalyze the CO_2_ hydration process (CO_2_ + H_2_O ↔ HCO^−^_3_ + H+). Numerous physiological processes are regulated by CAs, including the production of stomach acid, iron transport, homeostasis, bone resorption, renal acidification, gluconeogenesis and, respiration [16,17]. Many different investigations have demonstrated the significance of several members of the CA family in various cancer types. Specifically, it has been shown that CAIX acts as a biomarker for colon cancer as well as numerous other malignancies [18,19,20,21,22]. It has been demonstrated that colon cancer contains hypermethylated CAIV [23]. Hepatocellular carcinoma (HCC) has been reported to have reduced levels of CAI, CAII, and CAIII; this reduction has been shown to promote tumor cell motility and to contribute to tumor growth and metastasis [24]). Studies have drawn attention to the relationship between CAIII and cancer. Especially in liver cancer (HCC), it directs cells to invasion via the FAK signaling pathway with the intracellular/extracellular acidification mechanism. In this process, it has been shown that cells with significantly reduced CAIII expression increase their metastasis and invasion capabilities [25]. In addition, Türkoğlu et al showed in their study that TGF-β-induced CAIII expression in HT-29 cells, a colon cancer cell line, was suppressed via MEK-1 and PI3K pathways, and the same effect was also shown in transfection studies performed on deletion promotor constructs where TGF-β cytokine suppressed CAIII regulation. As a result, TGF-β, which increases ROS in cells in both colon cancer and osteosarcoma cells, contributed to the carcinogenesis process by causing a decrease in CAIII expression, which plays a role in the mechanism of protecting the cell from ROS [26]. In the literature searches, no information could be obtained regarding the regulation of CAIII in EGF-affected colon cancer. Therefore, EGF-effective regulation of CAIII has been investigated in both colon cancer models and non-carcinoma cell models. In our studies, it was found that EGF-effected CAIII regulation decreased at the mRNA and protein levels.

## 2. Materials and Methods

### 2.1. Cell Lines and Cell Culture

HT29, SW480, and HUVEC cells were cultured in high-glucose Dulbecco’s Modified Eagles Medium (DMEM, Sigma, St. Louis, MO, USA) containing 10% fetal calf serum (FCS; Sigma) and 1% L-Glutamine (Sigma) at 37 °C incubators with 5% CO_2_ in the air.

### 2.2. MTT Assay

3-(4,5-Dimethylthiazol-2-yl)-2,5-diphenyltetrazolium bromide (MTT) assay was used to analyze cell proliferation. SW480, HT29, and HUVEC cells were plated out of 96-well plates (5 × 10^4^ cells/well). Then, 20 ng EGF (Peprotech EC Ltd, London, England) was applied to the cells after 24 h of serum starvation for 24, 48, and 72 h. Untreated (NT) cells were used as control. The absorbance was determined at 550 nm using a spectrophotometric microplate reader (Thermo Scientific, Waltham, MA, USA) [27]. The assay was performed at least triplicate. Data was represented as fold value. Fold value was estimated using the following equation: OD550 (EGF-treated cells)/OD550 (Not treated (NT) cells).

### 2.3. RNA Isolation and Quantitative Real Time-PCR (qRT-PCR) Analysis

The GeneJET RNA isolation kit was used for total RNA isolation (Thermo Fisher Sci.). The recommended protocol was applied for RNA isolation from the HT29, SW480, and HUVEC cells. First, 1 μg of total RNA was reverse transcribed into cDNA using Revert Aid Reverse Transcriptase (Thermo Fisher Sci.) and Oligo (dT) as a primer. Real-time PCR was performed using 5 μL of RealQ Plus 2× Master Mix Green (Ampliqon) and 0.5 μL of each primer pair (10 ng/μL) in a final volume of 10 μL. The primer sequences for CAIII and β2M (β-2 microglobulin) gene were shown in Table S1. Each sample was assayed in triplicate, and the Ct value was calculated by the instrument (Light Cycler 485, Roche Molecular Biochemicals, Indianapolis, IN, USA). The relative change in gene expression between the control and the EGF-treated cells for each incubation time point was calculated according to 2^−ΔΔCt^ [28]. Human β-2 microglobulin (hβ-2) was used as an internal control. Fold changes in the CAIII mRNA expression were calculated by dividing 2^−ΔΔCt^ value of the EGF-treated cells by control cells.

### 2.4. Extraction of Proteins and Western Blotting

The radio immunoprecipitation assay (RIPA) buffer was used to obtain protein from the cells [29]. Protein concentration was measured based on the fluorimetric method using Qubit Protein assay kits (Thermo Fisher Sci.) and a Qubit Fluorometer. First, 50 μg of protein was loaded onto sodium dodecyl sulfate–polyacrylamide gel electrophoresis (SDS-PAGE). After proteins were transferred to a membrane (Immobilon-P PVDF, Millipore, Osaka), the membrane was incubated with CAIII- (Invitrogen, Carlsbad, CA, USA, PA525977) or β-Actin- (Santa Cruz Biotechnology, Santa Cruz, CA, USA, sc47778) specific antibodies. Secondary antibodies were then applied. Specific protein bands were treated and visualized with a chemiluminescence detection kit (ECL, Pierce, USA). Image J software (version 1.8.0) was used for densitometric analysis [30]. Fold changes in the CAIII protein expression were calculated by dividing the densitometry value of the EGF-treated cells by the control cells.

### 2.5. Statistical Analysis

Mini Tab 14 software was used to estimate the standard deviations and *p* values (Minitab, LLC., State College, PA, USA). One-way Anova analysis was used to determine the statistically significant differences between the pairs. *p*-value < 0.05 considered statistically significant [31].

## 3. Results

### 3.1. EGF Decreases CAIII mRNA Expression Level in Colon Cancer and Non-Cancer Cell Lines

**Figure 1:**
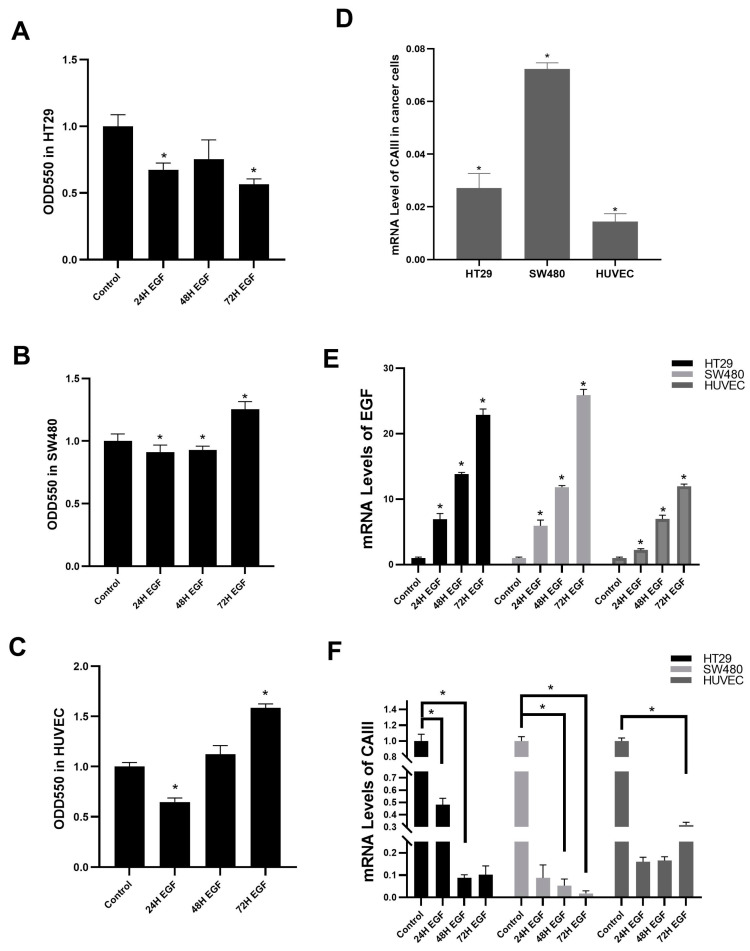
Effect of 20 ng EGF on CAIII. (**A**) Cell viability of HT29 cells after 20 ng EGF treatment. (**B**) Cell viability of SW480 cells after 20 ng EGF treatment. (**C**) Cell viability of HUVEC cells after 20 ng EGF treatment. (**D**) The mRNA level of CAIII was determined in different cancer cell lines (HT29, SW480 and HUVEC). (**E**) The mRNA level of EGF was determined in different cancer cell lines after 20 ng EGF treatment (HT29, SW480 and HUVEC). (**F**) The mRNA level of CAIII was determined in different cancer cell lines after 20 ng EGF treatment (HT29, SW480 and HUVEC). (* *p* ≤ 0.05).

We isolated total RNA from HT29 and SW480 cells as well as from the non-carcinoma model HUVEC cells in order to determine the presence of CAIII in the colon cancer models. Cultured cells were used to produce cDNAs, which were then amplified using particular primers for CAIII and the normalizing gene Human β-2 microglobulin (β2M). Colon cancer cells and non-cancer cell exhibited expression of CAIII mRNA, according to a qRTPCR study (Figure 1D). Using EGF-specific primers, real-time PCR was carried out to confirm the EGF-effective model. After 72 h of EGF treatment, it was found that the EGF mRNA level increased by about 25-fold in HT29 and SW480 cells and by 10-fold in HUVEC cells (Figure 1B). The MTT assay was run at 24, 48, and 72 h to determine the effect of EGF on the proliferation of HT29, SW480, and HUVEC cells. Normoxic control cells were untreated at these periods. After EGF treatment, it was found that there was no apparent cell proliferation in HT29, SW480, or HUVEC cells (*p* > 0.05) (Figure 1A–C).

In order to show the effect of EGF-effective conditions on CAIII mRNA expression, qRT-PCR was used using CAIII-specific primers. β2M primers were used as an internal control. Total RNAs from EGF treatment and non-treatment (Control Group) conditions were used for cDNA synthesis. mRNA expression of the CAIII level under EGF-effective conditions shows that CAIII has decreased in time-dependent in HT29, SW480, and HUVEC cells (Figure 1F). The CAIII mRNA level decreased specifically in 48 h and 72 h in the HT29 cell line under the EGF-effective conditions. In the SW480 cells, CAIII expression decreased at the early stages of EGF treatment. In the non-cancer model, HUVEC cells showed a similar response as the HT29 and SW480 cells, but the effect was not strong. In conclusion, these results show that in the CAIII mRNA regulation of EGF-effective mRNA, the level of CAIII expression is cell- and tissue-dependent.

Comparing the results of the analysis of the EGF-applied groups and the control group, it was determined that the expression of CAIII in the SW480 cells decreased more than the expression in HT-29 cells. The SW480 cell line is a grade 3–4 colon cancer cell model, while HT-29 is grade 1. These data show that the differences in the metastatic, invasive, and anti-apoptotic mechanisms of the cancer cell also affect the expression of CAIII.

### 3.2. EGF Decreases CAIII Protein Expression Level in Cancer and Non-Cancer Cell Lines

**Figure 2:**
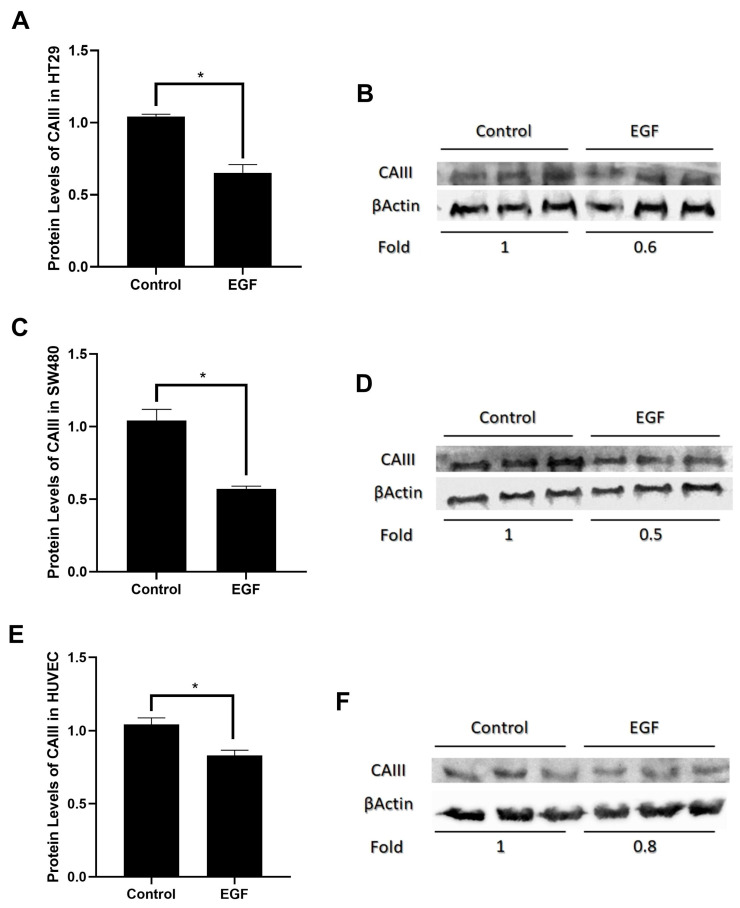
Effect of 20 ng EGF on CAIII protein expression level. (**A**) Protein level of CAIII on HT29 cell line after 20 ng EGF treatment. (**B**) CAIII protein expression was analyzed by Western blotting. Total cell lysates were prepared from EGF-treated and untreated HT29 cells at 48 h. (**C**) Protein level of CAIII on SW480 cell line after 20 ng EGF treatment. (**D**) CAIII protein expression was analyzed by Western blotting. Total cell lysates were prepared from EGF-treated and untreated SW480 cells at 48 h. (**E**) Protein level of CAIII on HUVEC cell line after 20 ng EGF treatment. (**F**) CAIII protein expression was analyzed by Western blotting. Total cell lysates were prepared from EGF-treated and untreated HUVEC cells at 48 h. (* *p* ≤ 0.05).

The Western blot technique was used to determine the expression of the increase in CAIII mRNA regulation at the protein level. Western blotting was performed using rabbit anti-CAIII or β-actin antibodies on cell lysates extracted from both EGF-treated and untreated cell pellets. Densitometric analysis was performed using the Image J program. Western blotting demonstrated that EGF lowered the quantity of CAIII protein in HT29, SW480, and HUVEC cells, which is consistent with the qRT-PCR results. It was evident that the HT29 cells’ CAII protein regulation was effectively decreased by EGF (Figure 2A,B). However, the effect was mostly determined in the SW480 cells (Figure 2C,D). The downregulation of CAIII in the HUVEC cells was less remarkable compared to the HT29 and SW480 cells (Figure 2E,F).

The decrease in CAIII expression determined in the mRNA data was also shown at the protein level. The difference in the decrease in the SW480 and HT-29 cell lines was also shown at the protein level. It caused the gene regulation of the cell character to change transcriptionally and translationally or to become more remarkable.

## 4. Discussion

The aim of the present study was to determine the effect of EGF cytokines on CAIII regulation in colon cancer cell lines. The cytotoxic effect of this cytokine was analyzed in colon cancer models, HT-29 and SW480, and a non-cancer model, HUVEC cells, to which 20 ng EGF was applied. It was determined that the EGF cytokine application did not cause a toxic condition in the cells. Real-time PCR and Western blot experiments were performed to determine how EGF cytokine regulates CAIII mRNA and protein expression in these cell lines in a time-dependent manner. As a result, it was determined that EGF cytokine suppresses CAIII regulation.

Cytosolic CAIII protein, a member of the CA family, draws attention with its low hydratase activity, unlike other members of the family. Studies have shown that CAIII increases the invasion of cells via the FAK signaling pathway, through intracellular or extracellular acidification. It has been shown that CAIII expression significantly decreases while increasing the metastasis and invasion abilities of cells [25]. When the expression level of CAIII in human hepatocellular carcinoma (HCC) was examined, it was observed that it decreased compared to the expression level in normal tissue, such as CAI and CAII, and the acidic pH increased in the extracellular and intracellular matrix with the overexpression of CAIII. The low pH level activated the focal adhesion kinase (FAK) pathway, which plays an important role in cell viability, migration, and spread [32,33].

The EGF-effective regulation of some carbonic anhydrase members has been shown in several studies. In these studies, it was determined that the CAIX expression level in breast cancer cells increased remarkably when EGF was applied [34]. The level of CAIX mRNA levels increased in U373 cell lines when induced by EGF, and that the upregulation of CAIX by EGF treatments is mediated through the activation of the MAPK and/or PI3K pathway [35]. In fact, Dorai and others have demonstrated that the tyrosine moiety of CAIX present in its intracellular domain can be phosphorylated in an EGF-dependent manner in the renal cell carcinoma cell line SKRC-01 [36].

Humoral factors, such as basic fibroblast growth factor (b-FGF) and epidermal growth factor (EGF), are necessary to activate the proliferation of intestinal stem/progenitor cells and to prevent their death [37,38]. EGFR is essential for the upkeep, growth, and self-renewal of cancer stem cells in numerous additional solid tumors [39,40]. EGF stimulates proliferation and directly or indirectly inhibits apoptosis by activating the PI3K/Akt signaling pathway in undifferentiated epithelial cells in the embryonic and adult intestines [41]. A poor prognosis has been linked to 60–80% of colonic tumors with overexpressed EGF receptor (EGFR) [42]. Through cell cycle dysregulation and increased cancer cell survival, the interaction of EGFR with TGFα and EGF promotes tumorigenesis [43]. Additionally, it has been demonstrated that TGFα interaction with EGFR promotes VEGF expression, and that higher serum levels of VEGF are linked to a shorter disease-free survival in patients with colorectal cancer; this could be because VEGF-mediated angiogenesis occurs through the migration, differentiation, and proliferation of vascular endothelial cells [43,44,45]. HGF’s capacity to stimulate angiogenesis in tumors is probably the reason why elevated blood levels of the hormone have been linked to the advancement of cancer, particularly colorectal cancer [46,47]. Studies have found that serum EGF levels are significantly higher in patients with early and late-stage CRCs as compared to healthy controls [48].

In order to determine the expression profile of CAIII, its expression levels in HT29, SW480, and HUVEC cell lines were examined. The effective regulation by EGF was examined in these cell lines where CAIII was determined to be expressed. It was determined that the regulation of CAIII, which has been determined to be associated with cancer, decreases in colon cancer cells under the influence of the EGF cytokine. EGF, which plays an active role in proliferation, induced the differentiation of the cells by inhibiting apoptosis. EGF-affected CAIII mRNA expression was suppressed in the colon cancer cell lines HT29 and SW480 and in the non-cancer cell line HUVEC cells. The difference between the two different selected colon cancer cell lines would be their cancer stages. The HT-29 cell line is grade 1 and SW480 is grade 3–4. Therefore, differences in the decrease in the regulation of the CAIII gene in the two different cell lines are remarkable. Especially in the SW480 cell line, the cell differentiation, invasion, and metastasis abilities are higher. It shows a more aggressive growth compared to the HT-29 cell line. The EGF cytokine regulates many biological factors, such as gene expression, cellular proliferation, angiogenesis, and apoptosis, which contribute to the development of malignancy in cells through the Ras-Raf mitogen-activated protein kinase pathway and the phosphatidyl inositol 3′ kinase and Akt pathway [49,50,51,52]. Additionally, EGF has been shown to support tumor cell motility, adhesion, and metastasis [52,53]. When all these are examined carefully, CAIII, which regulates the acidification of cells during the cancer progression process, aims to limit the ability of cancer cells to metastasize and invade. However, considering the effective regulation of EGF, the inhibition of CAIII revealed that the cells take on a more proliferative and aggressive character. It was determined that the role of EGF in regulating the development of malignancy was also expressed through CAIII, and that EGF-induced malignancy increased with the suppression of CAIII, which regulated intracellular/extracellular acidification during the cancerization process. Again, through protein analysis, our studies have shown that CAIII is also inhibited at the protein level in EGF effective regulation. This is evidence that the decrease in expression level is both transcriptional and translational. It has been shown that when TGF-B was applied to the HT-29 cells, a colon cancer cell line, the expression of CAIII at the mRNA and protein levels decreased. This decrease in CAIII regulation was due to the co-stimulation of TGF-B and EGF on EGFR [26].

In conclusion, in colon cancer cells, EGF contributes to the carcinogenesis process by reducing mRNA and protein CAIII expression, which plays a role in cell proliferation, angiogenesis, and apoptosis mechanisms. These data obtained with CAIII–EGF regulation are informative for future studies in determining through which signaling pathway EGFR regulates CAIII expression. In particular, changes related to cancer grade can be detailed at the cellular level, providing hope for patients with different grades of cancer.

## Data Availability

The data presented in this study are available on request.

## Supplementary Materials

The following supporting information can be downloaded at: https://www.mdpi.com/article/10.3390/cimb46110774/s1, Table S1: Sequences of primers used for PCR.
