# Supplementary material for: Epidermal Growth Factor Downregulates Carbon Anhydrase III (CAIII) in Colon Cancer
Source: Curr Issues Mol Biol. 2024 Nov 14;46(11):12994–3002. doi: 10.3390/cimb46110774 (PMC11593170; doi:10.3390/cimb46110774)
Supplement: Supplementary file 1 [file cimb-46-00774-s001.zip › cimb-3296513-supplementary.pdf]

Table S1. Sequences of primers used for PCR

Name	Sequence (5′-3′)
CAIII Forward	ACC ACT GGC ATG AAC TTT TCC CAA A
CAIII Reverse	TCA GAG CCA TGA TCA TCC GAA GAG C
β2M Forward	TTT CTG GCC TGG AGG CTA TC
β2M Reverse	CAT GTC TCC ATC CCA CTT AAC T
